# On Bathwater, Babies, and Designing Programs for Impact: Evaluations of the Integrated Community Case Management Strategy in Burkina Faso, Ethiopia, and Malawi

**DOI:** 10.4269/ajtmh.94-3intro1

**Published:** 2016-03-02

**Authors:** Elizabeth Hazel, Jennifer Bryce, the IIP-JHU iCCM Evaluation Working Group

**Affiliations:** Institute for International Programs, Johns Hopkins University, Baltimore, Maryland

The adage “don't throw the baby out with the bathwater” advises us not to discard the essential along with the unessential, whether due to impatience or frustration.^1^ This issue of the journal includes reports on prospective evaluations of the integrated community case management (iCCM) of childhood illness strategy in Burkina Faso,^2^ Ethiopia,^3^ and Malawi.^4^ iCCM seeks to reduce child mortality by making effective treatment of pneumonia, diarrhea, and malaria available from trained health workers at community level.^5^ In this commentary, we show results from the three countries, highlighting hard-won lessons about iCCM's potential to prevent unnecessary deaths among children.

The independent iCCM evaluations were designed in 2009–2010, as a part of the Catalytic Initiative to Save a Million Lives^6^ spearheaded by Canada and involving the Bill & Melinda Gates Foundation, the U.K. Department for International Development, the United Nations Children's Fund, the World Health Organization, the Doris Duke Charitable Foundation, and others. The goal of the Catalytic Initiative was to support and measure the impact of strong, coordinated efforts to deliver high-impact interventions to reduce under-five mortality in low-income, high-burden countries, with a particular focus on providing treatment for childhood pneumonia, diarrhea, and malaria at community level using iCCM.

The evaluation in each setting was conducted in collaboration with local research institutions, based on a locally adapted version of the common evaluation framework for maternal, newborn, and child health (MNCH).^7^ The evaluation designs were built on impact models reflecting the pathway to child survival. Beginning with policy and program inputs, the pathway moves to project implementation and intermediate outputs such as improved provision of quality services, strengthened health systems, and increased utilization. Continuing to coverage and behavioral outcomes, the pathway culminates in impact of under-five and postneonatal mortality. We focus on mortality among children aged 2–59 months here, because the iCCM strategy did not include guidelines for children under 2 months of age. Detailed methods are described in the individual papers; Table 1 provides a summary. All sites documented program inputs and contextual factors that may have affected program implementation or effectiveness. The evaluation teams shared intermediate and final results with governments and partners as a basis for strengthening iCCM implementation.

Selected results across the three countries are shown in Table 2. We reanalyzed some variables to maximize comparability across countries; in some instances, results presented here are not included in the series papers.

iCCM was not associated with accelerated mortality declines in children aged 2–59 months during the 2- to 4-year evaluation periods in these three settings. The reasons for this lack of association likely varied across settings, as does the likelihood that these programs may achieve measureable impact after additional years of sustained implementation.

In Burkina Faso, iCCM implementation did not reflect current best practice for designing and implementing effective MNCH programs. The program relied on community health worker (CHW) lay volunteers. The initial training was organized as a “cascade” in which representatives from districts were trained and then requested to organize subdistrict-level training sessions to train over 3,000 CHWs in the space of a few months, with minimal support or supervision. The training included written materials even though many of the CHWs were illiterate. Systems for ensuring the availability of drugs and reinforcing health worker performance were not consistently in place at the time of training, or indeed throughout the evaluated implementation period. There were no systematic efforts to generate community demand for and utilization of iCCM services. Not surprisingly, then, the quality of iCCM service provision was poor, the services were grossly underutilized, and there was no improvement in intervention areas in the proportion of sick children who received correct treatment of their disease (Table 2). The Burkina Faso iCCM program will not have an impact unless it is redesigned to fully capacitate health workers and educate the community to increase demand. Greater attention to accountability and best practices in women's and children's health should help prevent similar examples of incomplete and therefore ineffective programs in the future.^12^

However, in both Malawi and Ethiopia, iCCM was implemented more strongly and achieved important intermediate outputs. In both settings, government policies were in place to support full implementation of the strategy, and high numbers of paid community-level workers were well trained and initially well equipped. The percent of children receiving correct treatment from CHW for iCCM illnesses was moderately high in Ethiopia (78%) and lower in Malawi (63%) (Table 2) but was at least as good as the care received in first-level health facilities.^11^,^13^ In both settings, unfortunately, children with more severe illnesses were less likely than those without life-threatening conditions to be managed correctly, pointing to continuing needs for refinement of the iCCM approach to improve and sustain health worker performance.^11^,^13^ Health systems supports for iCCM were quite strong in Ethiopia, particularly high levels of clinical supervision, which requires further investigation to generate best practice models. Careful monitoring was used to generate remedial actions when problems were identified. In Malawi, only 58% of iCCM workers received clinical supervision and only 38% reported no stock-outs of essential iCCM drugs in the previous 3 months.^4^ Although far from perfect, both Ethiopia and Malawi implemented iCCM strongly and at scale, providing an important foundation for continued improvement.

However, in both Ethiopia and Malawi, there was no change in overall care seeking for iCCM illnesses and no impact on mortality (Table 2). The evaluation results also make clear that the potential effectiveness of iCCM was capped by low utilization. At baseline in Ethiopia, only 23% of mothers took their sick child to any formal provider for care, and care seeking rates did not increase over time. In contrast, care seeking in Malawi was quite high at baseline and remained the same at endline. Care seeking from iCCM providers increased slightly in both settings, but at such low absolute proportions that no population impact could be expected. No matter how strong iCCM service provision may be, the strategy cannot save lives unless mothers and other caregivers take their children for care. Future iCCM programs must be designed with this in mind, and include strong, locally defined components designed to increase care seeking and utilization.

In Malawi, particularly, the results suggest that geographic targeting of iCCM based on physical access to fixed health facilities may no longer be effective, or at least not sufficient. iCCM in Malawi was implemented in “hard-to-reach” areas of each district, as defined by district management teams, based on distance to a health post. Before 2008, this appeared to make good public health sense, when overall coverage for treatment interventions was low. By 2010, however, there were no differentials in children's receipt of appropriate treatment based on mothers' reports of whether distance to a health facility was a barrier to family health care.^4^ The assumption that reaching the unreached is limited by geography is no longer true, at least in Malawi.

Returning to the bathwater and baby adage, the iCCM in 2015 can be considered as the baby and the challenges of fully implementing iCCM as the bathwater. Like a human infant, iCCM programs will require steadfast attention, investment, and developmental guidance to mature and achieve their mortality-reduction potential. iCCM is not the answer in all settings—where first-level facilities are accessible and are or can be improved to provide quality services, such as in urban settings, there may be no need for iCCM, unless it can be demonstrated that the strategy overcomes barriers to care seeking that go beyond physical access. In settings where iCCM is needed, further research, development, and investment will be required to ensure that implementation plans reflect an accurate understanding of “which” women and children are not using services, “how best” to reach and motivate them to seek care, and “what role” iCCM can play in this pathway to survival. Ongoing programs of evaluation and implementation research can continue to generate information about the bathwater and the parts of iCCM and its context that must be discarded or refreshed. Local capacity to collect and analyze relevant data is a prerequisite for generating essential knowledge and putting this knowledge to use in the service of women and children.

## Figures and Tables

**Table 1:** Summary of evaluation designs

Characteristic	Burkina Faso	Ethiopia	Malawi
Evaluation design	Quasi-experimental design: purposively selected intervention and randomly selected comparison districts	Randomized control trial: “woredas” (districts) randomized in intervention and comparison	Dose–response analysis for 28 districts
Research partner(s)	Institut Supérieur des Sciences de la Population	MELA Research PLC; Alliance for Better Health Services PLC	National Statistical Office; Chancellor College, University of Malawi
Intervention area for the evaluation	Nine districts (seven malaria and diarrhea only, two iCCM for the three illnesses)	16 woredas	National, all 28 districts
Comparison area	Seven districts	15 woredas	No comparison area
Baseline–Endline	2010–2013	2011–2013	2010–2014
iCCM implementation strength assessment	2013	2012	2013
iCCM quality of care assessment	2012	2012	2009
Mortality measurement	Under-five mortality estimated using the Lives Saved Tool (LiST)	Under-five and 2–59 months mortality rates measured through full birth history, endline survey	Under-five and 2–59 months mortality rates measured through full birth history, endline survey
Additional sub-studies	Qualitative study of iCCM utilization^8^	Qualitative study of iCCM utilization^9^	Qualitative study of health system supports for iCCM^10^

iCCM = integrated community case management.

**Table 2:** Cross-country comparison of evaluation findings

	Definition	Burkina Faso	Ethiopia	Malawi
Policy	Presence of national iCCM policy	Partial	Yes	Yes
Readiness	Score of CHW “readiness” based on supervision and continuous drug stocks in the previous 3 months (*n* = CHWs providing iCCM)*	1† (385)	2.3† (137)	1.6† (3,392)
Quality of care	Percent of children receiving correct treatment from CHWs for iCCM illnesses (*n* = sick children seen by iCCM CHW)‡	36 (668)	78† (174)	63^11^ (242)
Utilization	Estimated number of child contacts with an iCCM-trained CHW, per child per year	0.23†	0.26†	0.93†
Coverage	Change in coverage of care seeking from all formal providers for the three illnesses combined in iCCM intervention areas between baseline and endline (baseline and endline [*n* = sick children 2–59 months of age]; pp difference)	57% (4,244)	23% (657)	68% (7,607)
		54% (3,915)	29% (1,116)	68% (9,634)
		−3 pp†	+6 pp†	0 pp†
Mortality	Mortality reduction attributable to iCCM program	None	None	None

CHW = community health worker; iCCM = integrated community case management; pp = percentage points. All data is from the three country papers in this series unless referenced otherwise.

* On a scale of 0–3; 3 is highest level of implementation strength.

† Data represent new analysis done for this commentary. See Webannex A for additional details on calculations.

‡ Defined as correct treatment and dosage: antibiotics for pneumonia, antimalarial for fever/malaria, oral dehydration salts (+recommended home fluids in Ethiopia), and zinc (Burkina and Ethiopia only; zinc treatment was not available through the iCCM program at the time of the Malawi study) for diarrhea.

## References

[1] Ammer C (1997). The American Heritage Dictionary of Idioms.

[2] Munos M, Guiella G, Roberton T, Maïga A, Tiendrebeogo A, Tam Y, Bryce J, Baya B (2016). Independent evaluation of the Rapid Scale-Up program to reduce under-five mortality in Burkina Faso. Am J Trop Med Hyg.

[3] Amouzou A, Shaw B, Miller NP, Tafesse M, Mekonnen Y, Moulton LH, Bryce J, Black RE (2016). Effects of the integrated Community Case Management of Childhood Illness strategy on child mortality in Ethiopia: a cluster randomized trial. Am J Trop Med Hyg.

[4] Amouzou A, Hazel E, Heidkamp R, Marsh A, Mleme T, Munthali S, Park L, Banda B, Moulton LH, Black RE, Hill K, Perin J, Victora CG, Bryce J (2016). Independent evaluation of the integrated Community Case Management of Childhood Illness strategy in Malawi using a national evaluation platform design. Am J Trop Med Hyg.

[5] Marsh DR, Pagnoni F, Peterson S (2012). Introduction to a special supplement: evidence for the implementation, effects, and impact of the integrated Community Case Management strategy to treat childhood infection. Am J Trop Med Hyg.

[6] Foreign Affairs, Trade and Development Canada (2015). The Catalytic Initiative to Save a Million Lives.

[7] Bryce J, Victora CG, Boerma T, Peters DH, Black RE (2011). Evaluating the scale-up for maternal and child survival: a common framework. Int Health.

[8] Institut Supérieur des Sciences de la Population and Institute for International Programs, Johns Hopkins Bloomberg School of Public Health (2014). Enquête sur la mise en œuvre de la prise en charge communautaire et la qualité des soins offerts aux enfants malades par les agents de santé communautaire: rapport final.

[9] Shaw B, Miller NP, Tsui AO, Bryce J, Tafesse M, Surkan PJ (2015). Determinants of utilization of health extension workers in the context of scale-up of integrated community case management of childhood illnesses in Ethiopia. Am J Trop Med Hyg.

[10] Callaghan-Koru JA, Hyder AA, George A, Nsona H, Mtimuni A, Zakeyo B, Mayani J, Cardemil C, Bryce J (2013). Health systems supports for community case management of childhood illness: lessons from an assessment of early implementation in Malawi. BMC Health Serv Res.

[11] Gilroy KE, Callaghan-Koru JA, Cardemil CV, Nsona H, Amouzou A, Mtimuni A, Daelmans B, Mgalula L, Bryce J, CCM-Malawi Quality of Care Working Group (2013). Quality of sick child care delivered by Health Surveillance Assistants in Malawi. Health Policy Plan.

[12] United Nations Foundation (2015). UN Commission on Information and Accountability for Women's and Children's Health.

[13] Miller NP, Tafesse M, Hazel E, Legesse H, Degefie T, Victora CG, Black RE, Bryce J (2014). Integrated community case management of childhood illness in Ethiopia: implementation strength and quality of care. Am J Trop Med Hyg.

